# Editorial: Rheumatic Diseases and Infection

**DOI:** 10.3389/fmed.2022.941678

**Published:** 2022-06-01

**Authors:** Lingli Dong, Hisanori Umehara, Jixin Zhong

**Affiliations:** ^1^Department of Rheumatology and Immunology, Tongji Hospital, Tongji Medical College, Huazhong University of Science and Technology, Wuhan, China; ^2^Department of Medicine, Nagahama City Hospital, Nagahama, Japan

**Keywords:** infection, rheumatic disease, virus, autoimmune disease (AID), inflammatory

Rheumatic diseases are a collective of inflammatory, often autoimmune, conditions that affect joints, muscles, bones, and organs (1). Although the exact etiology is largely unknown, both genetic and environmental factors are involved in the process of rheumatic disease. Among various environmental factors, infection has long been considered as an important risk and trigger for rheumatic disease (2). For example, systemic lupus erythematosus (SLE) was associated with the infection of Epstein-Barr virus (EBV). However, it is also believed that some infections may provide protection on autoimmune rheumatic diseases, namely hygiene hypothesis (3). This theory is supported by the epidemiological and clinical data that the increase of autoimmune diseases including rheumatic disease and allergy in the Western countries is accompanied by the decrease of infections over the past decades. Despite recent advances on dissecting the connection between rheumatic disease and infectious disease, the exact role of infections in the pathogenesis of rheumatic disease is not fully understood. This Research Topic highlights recent advances in clinical and translational aspects of rheumatic disease and infection. We thank all the authors who contributed to this topic by sharing their relevant work.

In this Research Topic, Iwata and Tanaka reviewed the connection between viral infection and systemic lupus erythematosus. Infections, especially viral infections, have been associated with both genetic and environmental risk factors of SLE. For example, human endogenous retrovirus (HERV) infection may cause genetic predisposition to SLE, whereas EBV infection, as an environmental risk, is a common trigger of SLE. IIwata and Tanaka summarized the latest findings on the crosstalk between the virus and immune system during the development of SLE. By analyzing 74,422 patients with systemic autoimmune rheumatic diseases (SARDs) and 297,688 matched patients without SARDs from the 1997–2013 Taiwanese National Health Insurance Research Database, Chen et al. reported an association between dengue infection history and increased risk of SLE (OR, 4.55; 95% CI, 2.77–7.46; *p* <0.001), but not Sjogren's syndrome (SS), rheumatoid arthritis (RA), systemic sclerosis (SSc), dermatomyositis (DM), or polymyositis (PM).

In a single-center retrospective study, Yang et al. analyzed 97 SLE patients who were hospitalized due to pulmonary infections and reported that these patients had a high mortality (34.02%). Cardiopulmonary involvement and opportunistic infection were independent risk factors for mortality in SLE patients hospitalized for pulmonary infections Yang et al.. By investigating 204 newly diagnosed IIM patients in a prospective cohort, Huang et al. reported that the percentage of recent cytomegalovirus (CMV) infections in idiopathic inflammatory myopathy (IIM) patients who were positive for anti- melanoma differentiation-associated protein 5 (MDA5) antibody was much higher than that in IIM patients negative for anti-MDA5 antibody. Furthermore, MDA5+ patients who were positive for CMV DNA had a much higher mortality in 1 year follow-up period, when compared with those negative for CMV-DNA. Therefore, Infection not only participate in the pathogenesis of autoimmune rheumatic disease, but also contribute to the mortality in these patients.

Patients diagnosed with autoimmune rheumatic diseases rely on disease-modifying anti-rheumatic drugs (DMARDs) to control the progression of disease. Both DMARDs and dysregulated immune function may cause increased risks of infection in patients with autoimmune rheumatic disease. It has been shown that patients with rheumatic disease are more susceptible to Coronavirus Disease 2019 (COVID-19), an ongoing global pandemic caused by severe acute respiratory syndrome coronavirus 2 (SARS-CoV-2) (4–7). In this Research Topic, Gracia et al. reported that the cumulative incidence in patients with systemic autoimmune diseases was 5 times higher than general population in Spain and treatment with anti-TNF-alpha was associated with an increased risk of SARS-CoV-2 infection. However, in another study including 358 patients with inflammatory arthritis receiving DMARDs, Favalli et al. found that the seroprevalence and titer of SARS-CoV-2 antibodies in patients with rheumatoid arthritis or spondyloarthritis in Lombardy area of Italy were comparable with the healthy population from the same region, although the use of glucocorticoids and comorbidities was associated with a higher seroprevalence rate in these patients.

Overall, the articles in this Research Topic highlight the importance of infections in autoimmune rheumatic diseases and provide us with a clearer insight into the interaction between infection and rheumatic disorders.

## Author Contributions

JZ wrote the manuscript. All authors have reviewed, edited the manuscript, and approved the submitted version.

## Funding

This work was supported by grants from the National Natural Science Foundation of China (Nos. 81974254 and 81771754).

## Conflict of Interest

The authors declare that the research was conducted in the absence of any commercial or financial relationships that could be construed as a potential conflict of interest.

## Publisher's Note

All claims expressed in this article are solely those of the authors and do not necessarily represent those of their affiliated organizations, or those of the publisher, the editors and the reviewers. Any product that may be evaluated in this article, or claim that may be made by its manufacturer, is not guaranteed or endorsed by the publisher.

## References

[1] Badii M Gaal O Popp RA Crisan TO Joosten LAB. Trained immunity and inflammation in rheumatic diseases. Joint Bone Spine. (2022) 89:105364. 10.1016/j.jbspin.2022.105364 35219890

[2] Gourley M Miller FW. Mechanisms of disease: Environmental factors in the pathogenesis of rheumatic disease. Nat Clin Pract Rheumatol. (2007) 3:172–80. 10.1038/ncprheum0435 17334340

[3] Bach JF. The hygiene hypothesis in autoimmunity: the role of pathogens and commensals. Nat Rev Immunol. (2018) 18:105–20. 10.1038/nri.2017.111 29034905

[4] Zhong J Shen G Yang H Huang A Chen X Dong L. COVID-19 in patients with rheumatic disease in Hubei province, China: a multicentre retrospective observational study. Lancet Rheumatol. (2020) 2:e557–e64. 10.1016/S2665-9913(20)30227-7 32838309 PMC7333992

[5] Grainger R Kim AHJ Conway R Yazdany J Robinson PC. COVID-19 in people with rheumatic diseases: risks, outcomes, treatment considerations. Nat Rev Rheumatol. (2022) 18:191–204. 10.1038/s41584-022-00755-x 35217850 PMC8874732

[6] Pablos JL Abasolo L Alvaro-Gracia JM Blanco FJ Blanco R Castrejon I. Prevalence of hospital PCR-confirmed COVID-19 cases in patients with chronic inflammatory and autoimmune rheumatic diseases. Ann Rheum Dis. (2020) 79:1170–3. 10.1136/annrheumdis-2020-217763 32532753 PMC7299645

[7] Shin YH Shin JI Moon SY Jin HY Kim SY Yang JM. Autoimmune inflammatory rheumatic diseases and COVID-19 outcomes in South Korea: a nationwide cohort study. Lancet Rheumatol. (2021) 3:e698–706. 10.1016/S2665-9913(21)00151-X 34179832 PMC8213376

